# Supplementary material for: Sex dependence of opioid-mediated responses to subanesthetic ketamine in rats
Source: Nat Commun. 2024 Jan 30;15:893. doi: 10.1038/s41467-024-45157-7 (PMC10828511; doi:10.1038/s41467-024-45157-7)
Supplement: Supplementary file 1 — Supplementary Information [file 41467_2024_45157_MOESM1_ESM.pdf]

Supplementary Information

Sex dependence of opioid-mediated responses to subanesthetic ketamine in rats

Tommaso Di Ianni[1#*], Sedona N. Ewbank[1], Marjorie R. Levinstein[2], Matine M. Azadian[1], Reece C. Budinich[2], Michael Michaelides[2,3], Raag D. Airan[1,4,5*]

[1] Department of Radiology, Stanford University School of Medicine, Stanford, CA 94305 USA

[2] Biobehavioral Imaging and Molecular Neuropsychopharmacology Unit, National Institute on Drug Abuse Intramural Research Program, Baltimore, MD 21224 USA

[3] Department of Psychiatry and Behavioral Sciences, Johns Hopkins University School of Medicine, Baltimore, MD 21205 USA

[4] Department of Materials Science and Engineering, Stanford University School of Medicine, Stanford, CA 94305 USA

[5] Department of Psychiatry and Behavioral Sciences, Stanford University School of Medicine, Stanford, CA 94305 USA

[#] Current address: Departments of Psychiatry & Behavioral Sciences and Radiology & Biomedical Imaging, University of California, San Francisco, San Francisco, CA 94114 USA

*Corresponding authors:

Tommaso Di Ianni, Ph.D.
Email: tommaso.diianni@ucsf.edu

Raag D. Airan, M.D., Ph.D.
Email: rairan@stanford.edu

Supplementary Tables 1, 2, 3

Statistical Analyses. Spreadsheets reporting the details of the statistical analyses with *P* values and effect sizes for all the figures can be found in Supplementary Tables 1, 2, and 3 at https://github.com/Airan-Lab/diianni2023-ketamine-fUSI/.

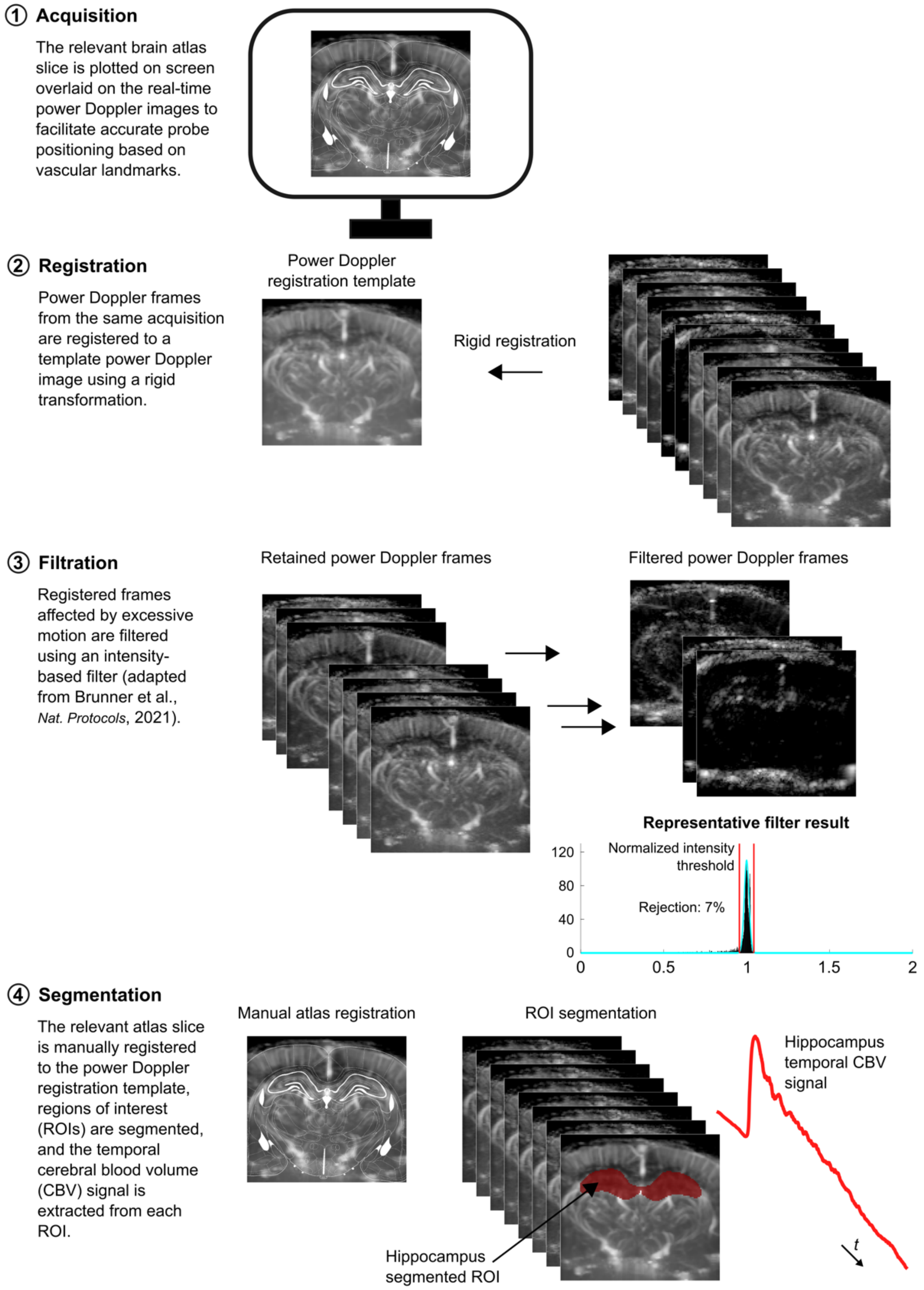

Supplementary Figure 1: Schematic representation of the power Doppler image registration and ROI segmentation procedure. A sub-sample of power Doppler images were plotted in real-time during the data acquisition ensure accurate probe positioning. The power Doppler images were then registered, filtered, and segmented offline to compute the ROI CBV signals. All the displayed power Doppler images are representative of a scan at bregma -3.5 mm used in this study.

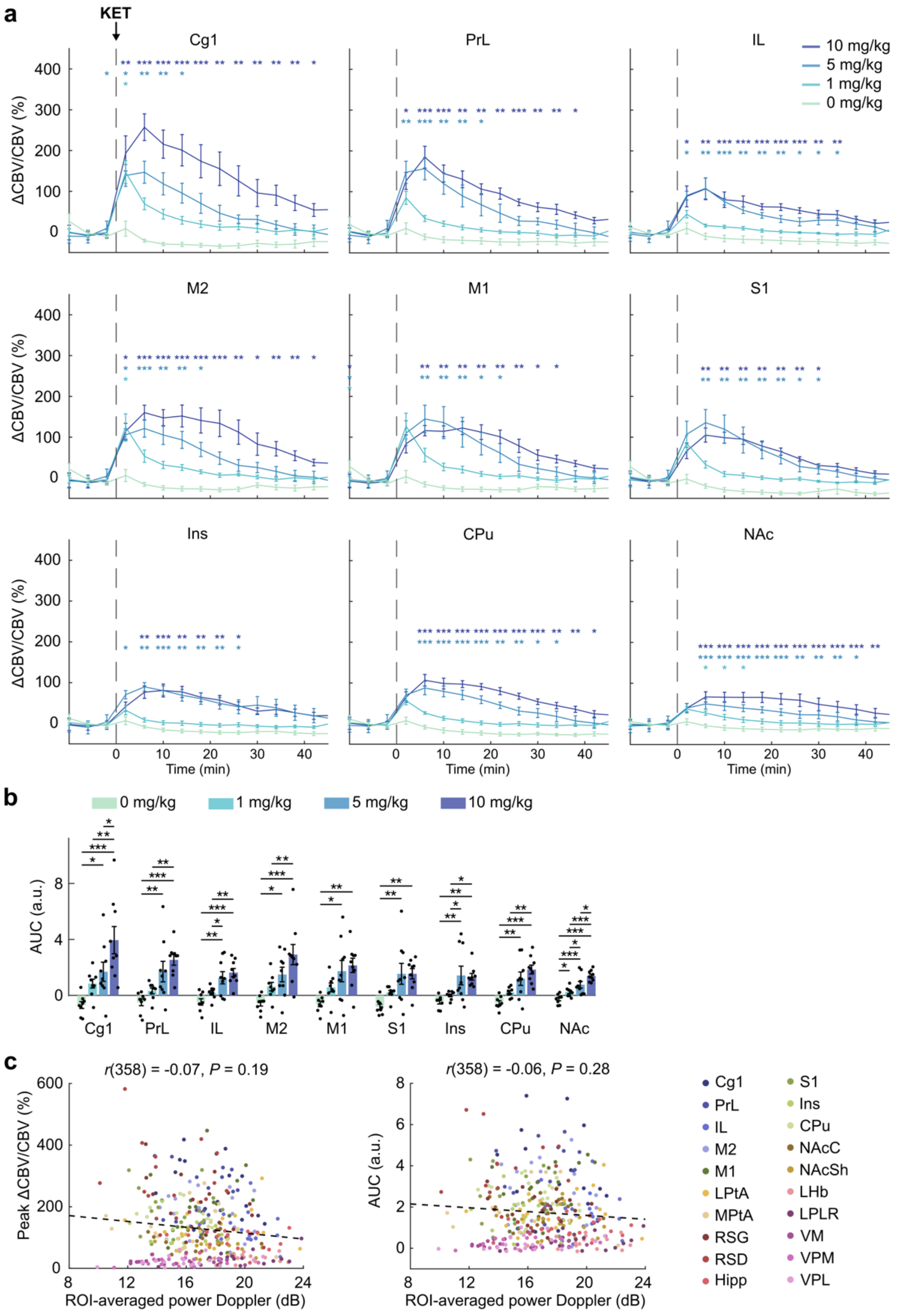

Supplementary Figure 2: Dose-dependent response of pharmacologic functional ultrasound imaging of ketamine induced responses. (**a**) Regional cerebral blood volume (CBV) signals in the segmented regions of interest were averaged in 4-min intervals for statistical analysis. Two-tailed unpaired *t*-test against the control group (0 mg/kg), *corrected *P* < 0.05, ***P* < 0.01, ****P* < 0.001. *n* =

9/group (10 and 5 mg/kg) or n = 8/group (1 and 0 mg/kg). (**b**) Area under the curve (AUC) of the CBV signals in the segmented ROIs with intravenous ketamine at increasing doses (0, 1, 5, and 10 mg/kg). Two-way mixed-effects ANOVA; within-subjects factor of region, $F_{2.78,83.35}$ = 5.32, P = 0.003; between-subjects factor of dose, $F_{3,30}$ = 10.47, P = 7.11E-05; interaction, $F_{8.33,83.35}$ = 2.62, P = 0.012. Two-tailed unpaired *t*-test, *corrected P < 0.05; **P < 0.01; ***P < 0.001. n = 9 rats/group (10 and 5 mg/kg); n = 8 rats/group (1 and 0 mg/kg). (**c**) Correlation of ROI-averaged power Doppler with regional peak CBV and regional AUC. Spearman's rank correlation, 360 ROI-segmented values from n = 36 rats. Data are presented as mean +/- SEM. Source data are provided as a Source Data file. Details on the statistical analyses are provided in Supplementary Table 1.

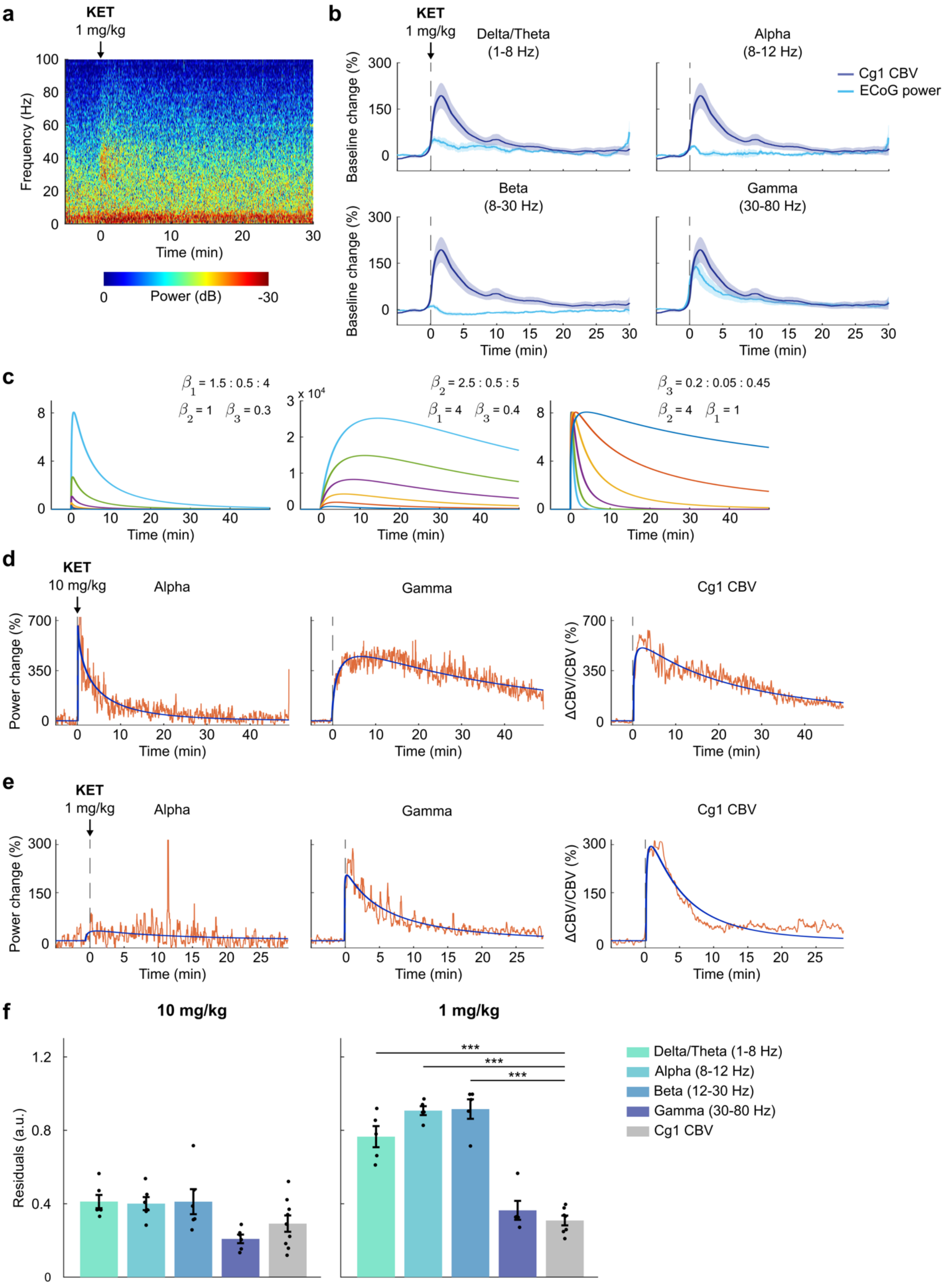
a
KET
1 mg/kg
Frequency (Hz)
Time (min)
Power (dB)
b
Delta/Theta
(1-8 Hz)
Alpha
(8-12 Hz)
Beta
(8-30 Hz)
Gamma
(30-80 Hz)
Cg1 CBV
ECoG power
Baseline change (%)
c
β_1 = 1.5 : 0.5 : 4
β_2 = 1 β_3 = 0.3
β_2 = 2.5 : 0.5 : 5
β_1 = 4 β_3 = 0.4
β_3 = 0.2 : 0.05 : 0.45
β_2 = 4 β_1 = 1
d
KET
10 mg/kg
Alpha
Gamma
Cg1 CBV
Power change (%)
ΔCBV/CBV (%)
e
KET
1 mg/kg
f
10 mg/kg
1 mg/kg
Residuals (a.u.)
Delta/Theta (1-8 Hz)
Alpha (8-12 Hz)
Beta (12-30 Hz)
Gamma (30-80 Hz)
Cg1 CBV

Supplementary Figure 3: Electrophysiology time series and regression using a four-parameter gamma-distribution function. (**a**) Representative spectrogram for i.v. administration of 1 mg/kg ketamine (KET). (**b**) Time series of normalized electrocorticography (ECoG) power changes in each frequency band and cerebral blood volume (CBV) signal in the Cg1 region in response to 1 mg/kg i.v. ketamine. Solid lines are the mean values and shaded areas are SEM. (**c**) Examples of gamma-distribution functions obtained by varying β_1, β_2, and β_3 in the equation of Fig. 2D within the reported range. (**d-e**) Representative ECoG/CBV signals and relative regression results for 10 mg/kg (**d**) and 1 mg/kg (**e**) i.v. ketamine. (**f**) Normalized least-squares minimization residuals resulting from the regression of ECoG/CBV signals for 10 mg/kg and 1 mg/kg i.v. ketamine. Two-tailed unpaired *t*-test comparing each band to CBV, ***corrected $P < 0.001$. ECoG: $n = 5$ rats for 1 mg/kg, $n = 6$ rats for 10 mg/kg; CBV: $n = 7$ rats for 1 mg/kg, $n = 9$ rats for 10 mg/kg. Data are presented as mean +/- SEM. Source data are provided as a Source Data file. Details on the statistical analyses are provided in Supplementary Table 1.

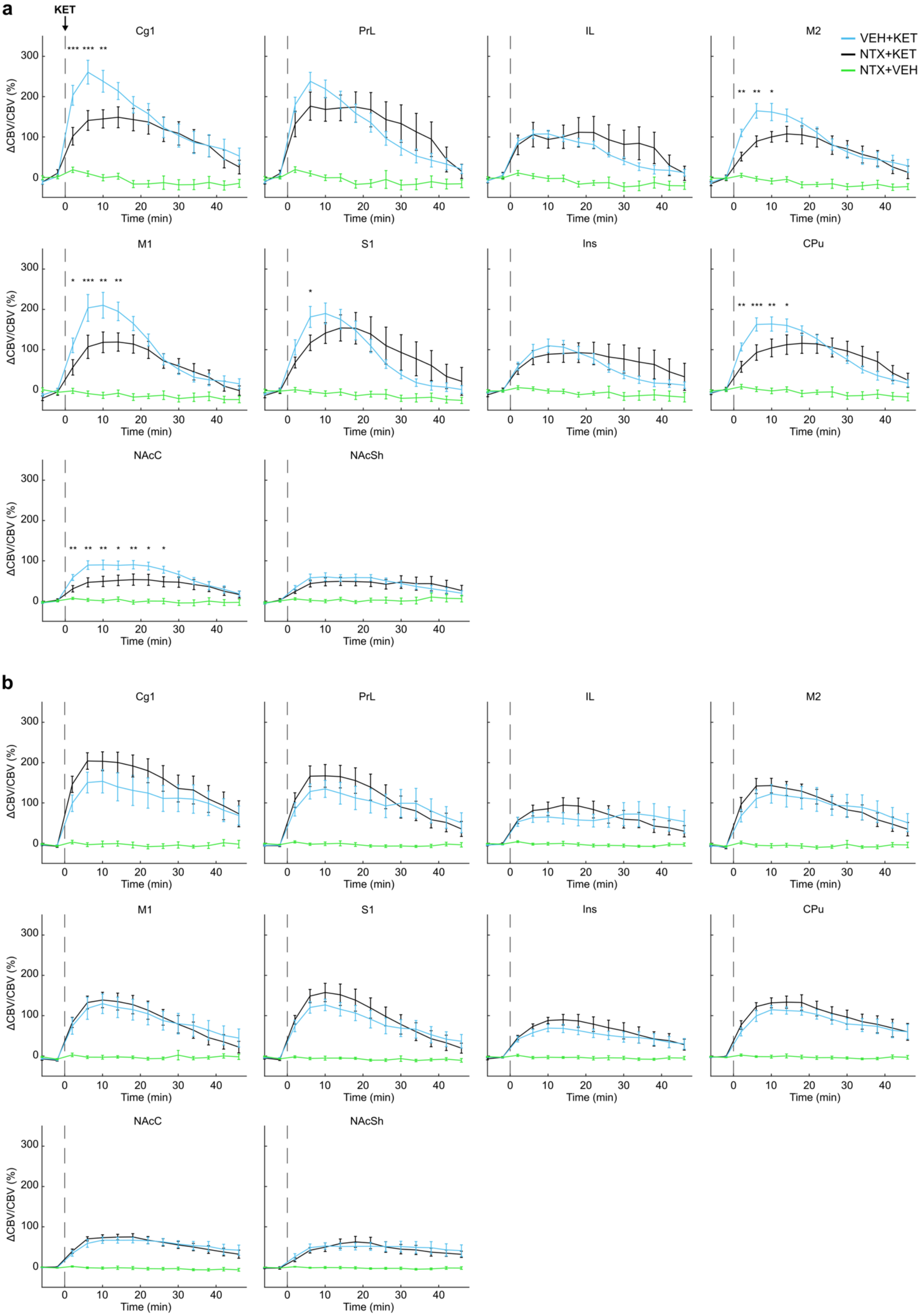
a
KET
Cg1
PrL
IL
M2
M1
S1
Ins
CPu
NAcC
NAcSh
VEH+KET
NTX+KET
NTX+VEH
ΔCBV/CBV (%)
Time (min)
b
Cg1
PrL
IL
M2
M1
S1
Ins
CPu
NAcC
NAcSh
ΔCBV/CBV (%)
Time (min)

Supplementary Figure 4: Regional cerebral blood volume (CBV) time series following ketamine administration at bregma +2.5 mm. (**a**) Regional CBV signals recorded in male rats and averaged in 4-min intervals. Two-tailed paired *t*-test, *corrected $P < 0.05$, **$P < 0.01$, ***$P < 0.001$. $n = 9$ male rats. Significance is only displayed for the VEH+KET vs NTX+KET comparison. (**b**) Regional CBV signals recorded in male rats and averaged in 4-min intervals. Two-tailed paired *t*-test comparing the VEH+KET vs NTX+KET groups showed no significant effects. $n = 9$ female rats. Significance is only displayed for the VEH+KET vs NTX+KET comparison. Data are presented as mean +/- SEM. Source data are provided as a Source Data file. Details on the statistical analyses are provided in Supplementary Table 1.

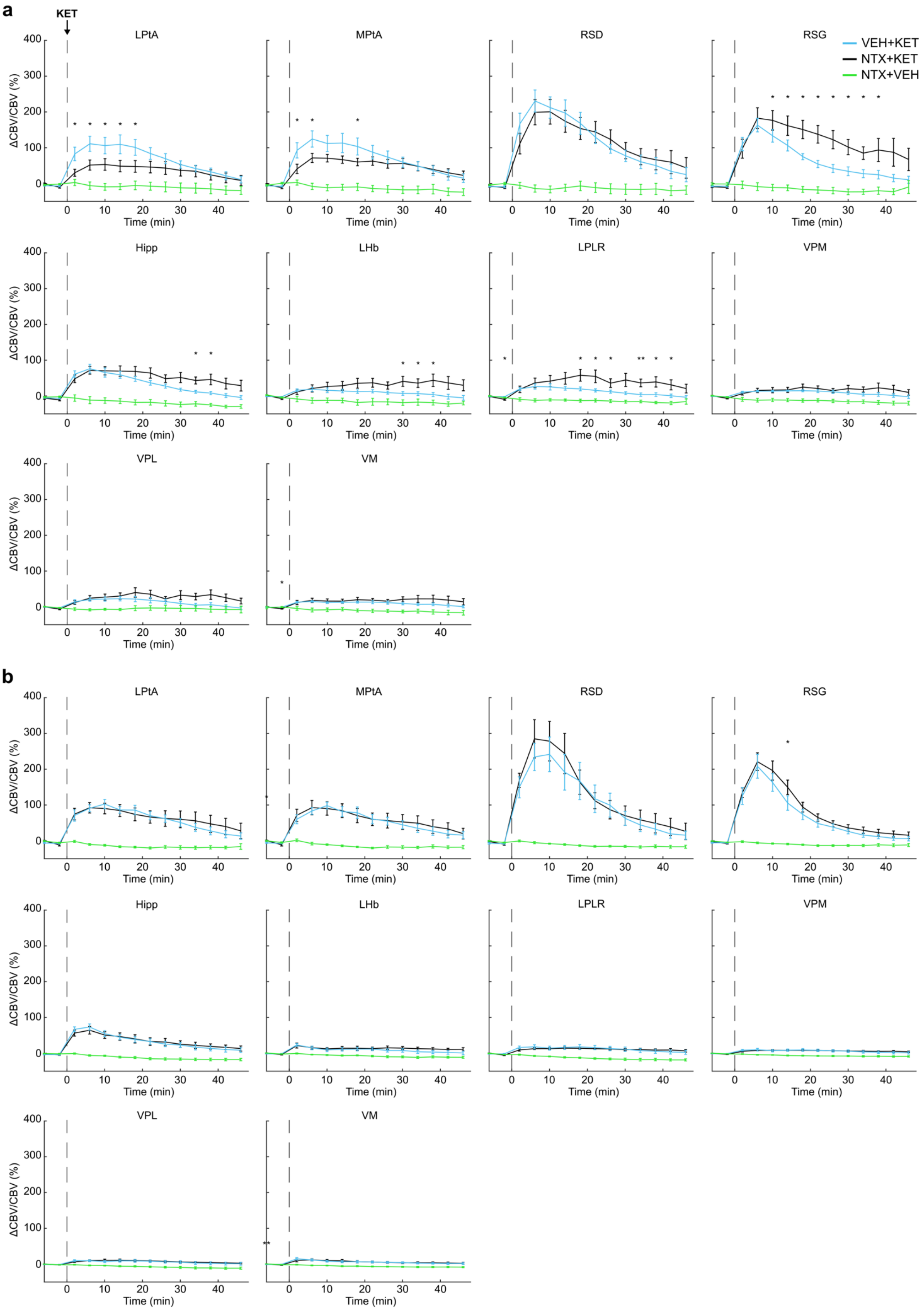
a
KET
LPtA
MPtA
RSD
RSG
VEH+KET
NTX+KET
NTX+VEH
Hipp
LHb
LPLR
VPM
VPL
VM
ΔCBV/CBV (%)
Time (min)
b
LPtA
MPtA
RSD
RSG
Hipp
LHb
LPLR
VPM
VPL
VM

Supplementary Figure 5: Regional cerebral blood volume (CBV) time series following ketamine administration at bregma -3.5 mm. (**a**) Regional CBV signals recorded in male rats and averaged in 4-min intervals. Two-tailed paired *t*-test, VEH+KET vs NTX+KET, *corrected $P < 0.05$, **$P < 0.01$. $n = 9$ male rats. Significance is only displayed for the VEH+KET vs NTX+KET comparison. (**b**) Regional CBV signals recorded in female rats and averaged in 4-min intervals. Two-tailed paired *t*-test, VEH+KET vs NTX+KET, *corrected $P < 0.05$, **$P < 0.01$. $n = 9$ female rats. Significance is only displayed for the VEH+KET vs NTX+KET comparison. Data are presented as mean +/- SEM. Source data are provided as a Source Data file. Details on the statistical analyses are provided in Supplementary Table 1.

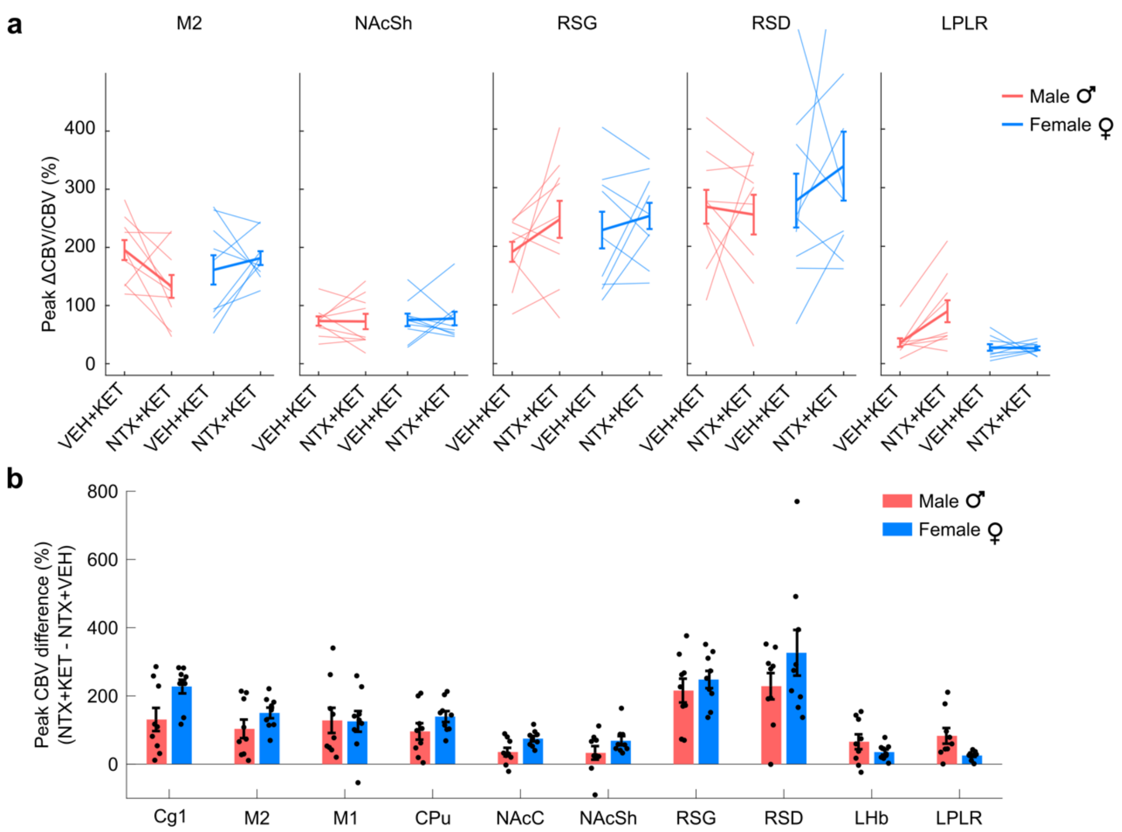

Supplementary Figure 6: Region-wise group-level analysis of subanesthetic ketamine effects. (**a**) Peak cerebral blood volume (CBV) changes in individual male and female rats receiving vehicle or naltrexone pretreatment. Two-tailed paired t-test (NTX+KET vs VEH+KET) or two-tailed unpaired *t*-test (F vs M). No significant effects were observed in these regions. Differences in LPLR in male rats were significant before multiple comparisons correction ($P = 0.012$; Hedge's $g = 0.97$). $n = 9$ male; $n = 9$ female. (**b**) Peak CBV differences between the NTX+KET and NTX+VEH treatments in individual rats were compared between males and females. One-way ANOVA for sex factor, $F_{1,178} = 3.22$, $P = 0.075$. Two-tailed unpaired *t*-test (M vs F) showed no significant differences (corrected $P > 0.087$). Data are presented as mean +/- SEM. Source data are provided as a Source Data file. Details on the statistical analyses are provided in Supplementary Table 1.

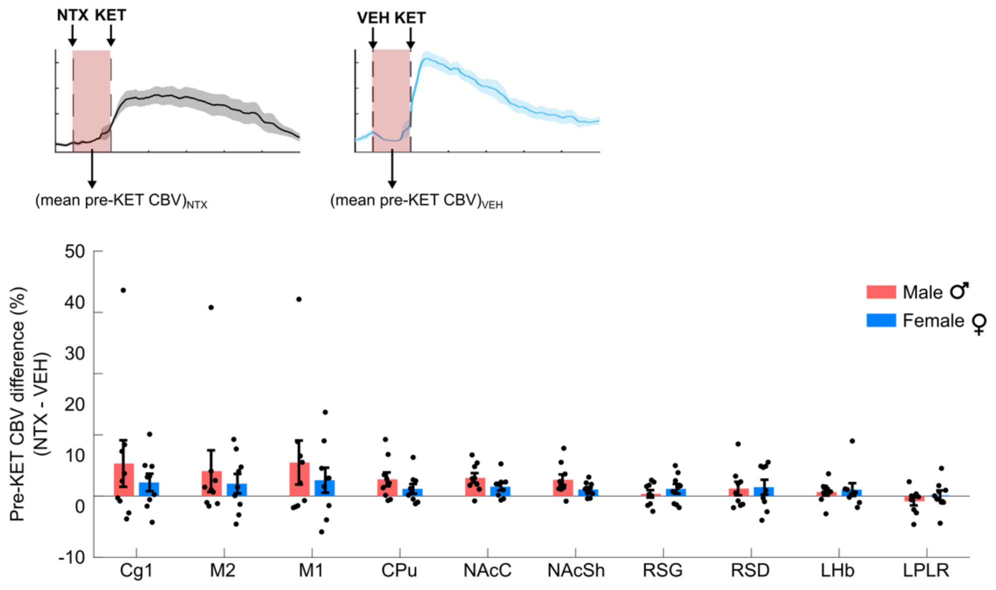

Supplementary Figure 7: Analysis of naltrexone-induced CBV changes during the pre-ketamine baseline period. Mean pre-ketamine CBV differences between the NTX+KET and VEH+KET treatment conditions in individual rats were compared between male and female subjects. One-way ANOVA for sex factor, $F_{1,178}$ = 1.83, P = 0.178. Two-tailed unpaired t-test (M vs F) showed no significant differences (corrected P > 0.71). Two-tailed paired t-test between regions showed no significant differences (corrected P > 0.49). Data are presented as mean +/- SEM. Source data are provided as a Source Data file. Details on the statistical analyses are provided in Supplementary Table 1.

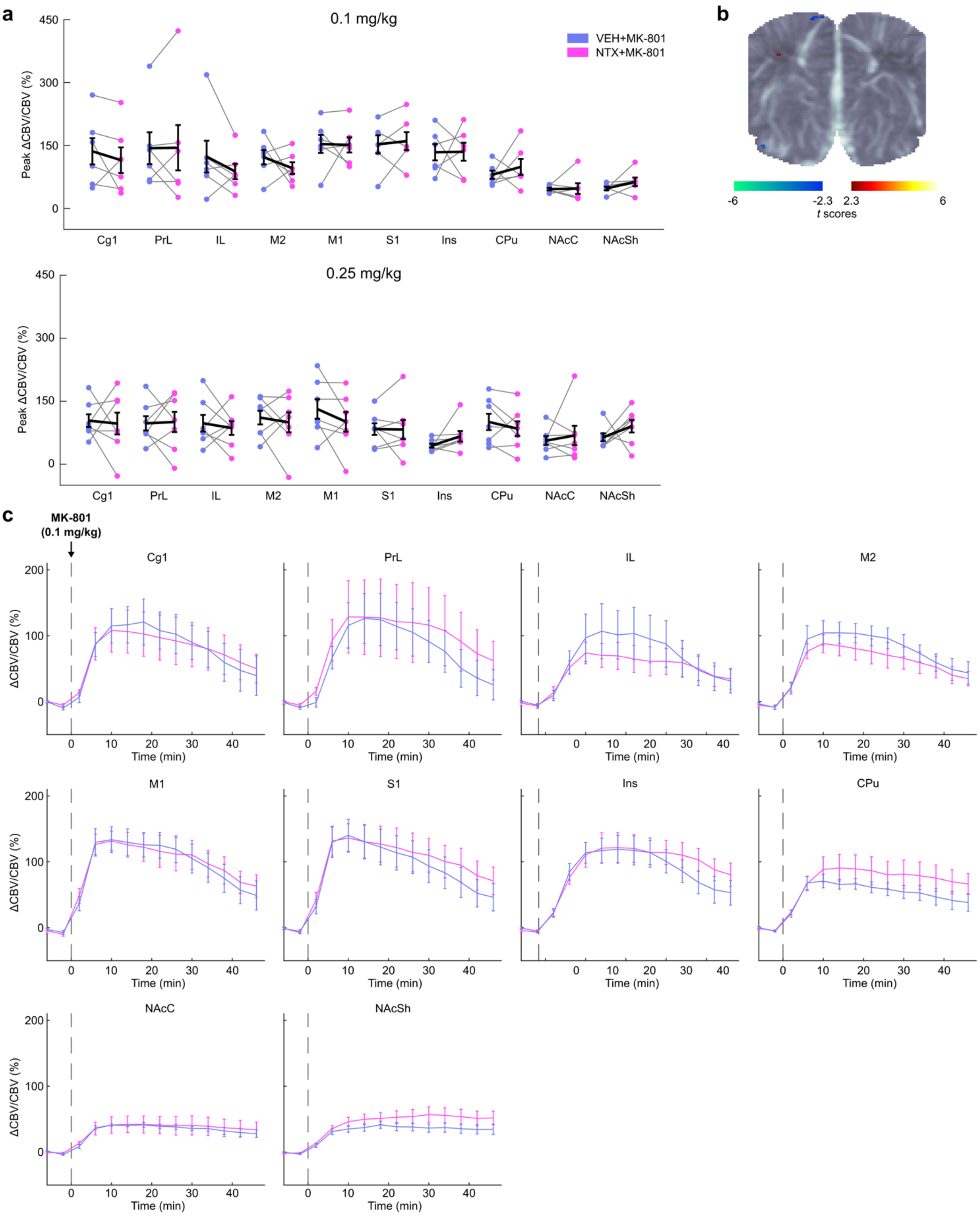

Supplementary Figure 8: Naltrexone pretreatment does not affect neural activity changes evoked by MK-801. Male rats were administered either vehicle (VEH) naltrexone (NTX; 10 mg/kg) followed by an injection of MK-801 (0.1 mg/kg or 0.25 mg/kg) after 10 min. Each animal was imaged two times under the treatment conditions of VEH+MK-801 and NTX+MK-801. (**a**) Peak cerebral blood volume (CBV)

changes in individual rats with 0.1 mg/kg and 0.25 mg/kg MK-801. 0.1 mg/kg: two-way ANOVA; within-subjects factor of region, $F_{9,45}$ = 4.08, P = 7.1E-04; within-subjects factor of treatment, $F_{1,5}$ = 0.05, P = 0.83; interaction, $F_{9,45}$ = 0.87, P = 0.56. Two-sided paired *t*-tests (VEH+MK-801 vs NTX+MK-801) showed no significant effects. n = 6 male rats. 0.25 mg/kg: two-way ANOVA; within-subjects factor of region, $F_{9,54}$ = 2.08, P = 0.048; within-subjects factor of treatment, $F_{1,6}$ = 0.02, P = 0.9; interaction, $F_{9,54}$ = 0.56, P = 0.8. Two-sided paired *t*-tests (VEH+MK-801 vs NTX+MK-801) showed no significant effects. n = 7 male rats (**b**) Functional map at bregma +2.5 mm with the 0.1 mg/kg MK-801 dose. The *t* scores were calculated by contrasting the pixel-wise peak cerebral blood volume (CBV) in the two treatment groups. The *t* scores were thresholded to show only the statistically significant pixels (two-sided paired *t*-test, $P < 0.05$). This map was not corrected for multiple comparisons. n = 6 male rats. (**c**) Regional CBV signals in the segmented ROIs averaged in 4-min intervals with the 0.1 mg/kg MK-801 dose. Two-tailed paired *t*-test showed no significant effects. n = 6 male rats. Data are presented as mean +/- SEM. Source data are provided as a Source Data file. Details on the statistical analyses are provided in Supplementary Table 1.
