# Supplementary material for: Sex dependence of opioid-mediated responses to subanesthetic ketamine in rats
Source: Nat Commun. 2024 Jan 30;15:893. doi: 10.1038/s41467-024-45157-7 (PMC10828511; doi:10.1038/s41467-024-45157-7)
Supplement: Supplementary file 5 — Description of Additional Supplementary Information [file 41467_2024_45157_MOESM5_ESM.pdf]

Description of Additional Supplementary Files

File Name: Supplementary Movie 1
Description: Brain-wide activity in two coronal planes following ketamine administration (10 mg/kg, intravenous). The cerebral blood volume (CBV) maps were calculated versus a pre-injection baseline and displayed overlaid on the respective power Doppler frames. Timestamp is in min:sec post ketamine.

File Name: Supplementary Movie 2
Description: Statistical maps comparing male rats administered ketamine (10 mg/kg, intravenous) and pretreated with the opioid receptor antagonist naltrexone (10 mg/kg, subcutaneous) (NTX+KET group) versus saline (VEH+KET group). The t scores for the two coronal planes are overlaid on the respective power Doppler frames. Timestamp is in min:sec post ketamine.
