# Supplementary material for: Sex dependence of opioid-mediated responses to subanesthetic ketamine in rats
Source: Nat Commun. 2024 Jan 30;15:893. doi: 10.1038/s41467-024-45157-7 (PMC10828511; doi:10.1038/s41467-024-45157-7)
Supplement: Supplementary file 6 — Reporting Summary [file 41467_2024_45157_MOESM6_ESM.pdf]

nature portfolio

Corresponding author(s): Tommaso Di Ianni, PhD
Raag Airan, MD PhD

Last updated by author(s): 2023/12/17

Reporting Summary

Nature Portfolio wishes to improve the reproducibility of the work that we publish. This form provides structure for consistency and transparency in reporting. For further information on Nature Portfolio policies, see our Editorial Policies and the Editorial Policy Checklist.

Statistics

For all statistical analyses, confirm that the following items are present in the figure legend, table legend, main text, or Methods section.

n/a	Confirmed	
☐	☒	The exact sample size (n) for each experimental group/condition, given as a discrete number and unit of measurement
☐	☒	A statement on whether measurements were taken from distinct samples or whether the same sample was measured repeatedly
☐	☒	The statistical test(s) used AND whether they are one- or two-sided<br>*Only common tests should be described solely by name; describe more complex techniques in the Methods section.*
☒	☐	A description of all covariates tested
☐	☒	A description of any assumptions or corrections, such as tests of normality and adjustment for multiple comparisons
☐	☒	A full description of the statistical parameters including central tendency (e.g. means) or other basic estimates (e.g. regression coefficient) AND variation (e.g. standard deviation) or associated estimates of uncertainty (e.g. confidence intervals)
☐	☒	For null hypothesis testing, the test statistic (e.g. F, t, r) with confidence intervals, effect sizes, degrees of freedom and P value noted<br>*Give P values as exact values whenever suitable.*
☒	☐	For Bayesian analysis, information on the choice of priors and Markov chain Monte Carlo settings
☒	☐	For hierarchical and complex designs, identification of the appropriate level for tests and full reporting of outcomes
☐	☒	Estimates of effect sizes (e.g. Cohen's d, Pearson's r), indicating how they were calculated

Our web collection on statistics for biologists contains articles on many of the points above.

Software and code

Policy information about availability of computer code

Data collection	Data collection was performed in MATLAB R2021.
Data analysis	Behavioral data were analyzed with ToxTrack v2.61. Immunohistochemistry images were analyzed with BZ-X Advanced Analysis Software. Autoradiography images were analyzed in ImageJ. All the statistical analyses were performed in R Studio 2022.07.01 and MATLAB R2021. Codes for all the statistical analyses are available at https://github.com/Airan-Lab/diianni2023-ketamine-fUSI/.

For manuscripts utilizing custom algorithms or software that are central to the research but not yet described in published literature, software must be made available to editors and reviewers. We strongly encourage code deposition in a community repository (e.g. GitHub). See the Nature Portfolio guidelines for submitting code & software for further information.

Data

Policy information about availability of data

All manuscripts must include a data availability statement. This statement should provide the following information, where applicable:

- Accession codes, unique identifiers, or web links for publicly available datasets
- A description of any restrictions on data availability
- For clinical datasets or third party data, please ensure that the statement adheres to our policy

The data generated in this study and used in all the statistical analyses are provided in the Source Data file. The raw functional ultrasound imaging data were not deposited due to the large size of each data acquisition. Access can be obtained upon reasonable request from the corresponding authors.

Research involving human participants, their data, or biological material

Policy information about studies with human participants or human data. See also policy information about sex, gender (identity/presentation), and sexual orientation and race, ethnicity and racism.

Reporting on sex and gender	N/A
Reporting on race, ethnicity, or other socially relevant groupings	N/A
Population characteristics	N/A
Recruitment	N/A
Ethics oversight	N/A

Note that full information on the approval of the study protocol must also be provided in the manuscript.

Field-specific reporting

Please select the one below that is the best fit for your research. If you are not sure, read the appropriate sections before making your selection.

☒ Life sciences ☐ Behavioural & social sciences ☐ Ecological, evolutionary & environmental sciences

For a reference copy of the document with all sections, see nature.com/documents/nr-reporting-summary-flat.pdf

Life sciences study design

All studies must disclose on these points even when the disclosure is negative.

Sample size	Sample sizes were determined based on our prior experience with pharmaco-fUSI (Levinstein et al., Biological Psychiatry, 2023) and previous published behavioral studies.
Data exclusions	In the functional imaging experiments, criteria for data exclusion were pre-established: data sets were excluded in case of excessive animal motion or evident signs of hypoxia during data acquisition. No data were excluded from the analysis of other experiments.
Replication	The presented data were collected over a large number of experimental sessions (depending on the reported sample sizes), in separate cohorts of rats and over a period extending several months for each experimental group. Behavioral experiments were replicated by a male and a female experimenter to control for experimenter's sex.
Randomization	In the functional imaging experiments where animals were imaged repeatedly (up to three times), treatment conditions were assigned in randomized order and counterbalanced within each experimental group. In all the other experiments, animals were randomly assigned to the treatment group.
Blinding	Experimenters were blinded to group allocation during data analysis. All data analyses and statistical comparisons were automated and performed in batch. Experimenters were not blinded to the administered drug during the imaging experiments.

Reporting for specific materials, systems and methods

We require information from authors about some types of materials, experimental systems and methods used in many studies. Here, indicate whether each material, system or method listed is relevant to your study. If you are not sure if a list item applies to your research, read the appropriate section before selecting a response.

Materials & experimental systems

n/a	Involved in the study	
☐	☒	Antibodies
☒	☐	Eukaryotic cell lines
☒	☐	Palaeontology and archaeology
☐	☒	Animals and other organisms
☒	☐	Clinical data
☒	☐	Dual use research of concern
☒	☐	Plants

Methods

n/a	Involved in the study	
☒	☐	ChIP-seq
☒	☐	Flow cytometry
☒	☐	MRI-based neuroimaging

Antibodies

Antibodies used	Rabbit monoclonal to PSD95 - Synaptic Marker(Abcam; ab238135). Goat-anti-rabbit Alexa Fluor 555 (Invitrogen; A-21428).
Validation	Zhang Y et al. Mdivi-1 alleviates brain damage and synaptic dysfunction after intracerebral hemorrhage in mice. Exp Brain Res 239:1581-1593 (2021). PubMed: 33754161<br>He C et al. Low-glucose-sensitive TRPC6 dysfunction drives hypoglycemia-induced cognitive impairment in diabetes. Clin Transl Med 10:e205 (2020).

Animals and other research organisms

Policy information about studies involving animals; ARRIVE guidelines recommended for reporting animal research, and Sex and Gender in Research

Laboratory animals	Long Evans rats; 9-10 weeks old
Wild animals	No wild animals were used in this study.
Reporting on sex	Male and female rats were used in this study. Statistical analyses considering sex as a biological factor were performed wherever appropriate. P values and effective sizes are reported in the main text and figure captions.
Field-collected samples	No field-collected samples were used in this study.
Ethics oversight	The experimental protocols for all the animal procedures was approved by the Institutional Animal Care and Use Committee at Stanford University and at the National Institute on Drug Abuse.

Note that full information on the approval of the study protocol must also be provided in the manuscript.

Plants

Seed stocks	N/A
Novel plant genotypes	N/A
Authentication	N/A
